# Retinal nerve fiber layer thickness and cognitive ability in older people: the Lothian Birth Cohort 1936 study

**DOI:** 10.1186/1471-2415-13-28

**Published:** 2013-07-03

**Authors:** Augustinus Laude, Gerassimos Lascaratos, Ross D Henderson, John M Starr, Ian J Deary, Baljean Dhillon

**Affiliations:** 1 Princess Alexandra Eye Pavilion, NHS Lothian, Scotland, UK; 2 National Healthcare Group Eye Institute, Tan Tock Seng Hospital, Singapore 308433, Singapore; 3 Centre for Cognitive Ageing and Cognitive Epidemiology, Department of Psychology, University of Edinburgh, Scotland, UK; 4 Centre for Cognitive Ageing and Cognitive Epidemiology, Geriatric Medicine Unit, University of Edinburgh, Scotland, UK; 5 School of Clinical Sciences, University of Edinburgh, Scotland, UK

**Keywords:** Cognitive, Elderly, Principal components analysis, Optical coherence tomography, Retinal nerve fiber layer

## Abstract

**Background:**

This study aims to examine the relationship between the retinal nerve fiber layer (RNFL) thickness as measured by optical coherence tomography (OCT) and lifetime cognitive change in healthy older people.

**Methods:**

In a narrow-age sample population from the Lothian Birth Cohort 1936 who were all aged approximately 72 years when tested, participants underwent RNFL measurements using OCT. General linear modeling was used to calculate the effect of RNFL thickness on three domains; general cognitive ability (g-factor), general processing speed (g-speed) and general memory ability (g-memory) using age at time of assessment and gender as co-variates.

**Results:**

Of 105 participants, 96 completed OCT scans that were of suitable quality for assessment were analyzed. Using age and gender as covariates, we found only one significant association, between the inferior area RNFL thickness and g-speed (*p* = 0.049, η^2^ = 0.045). Interestingly, when we included age 11 IQ as a covariate in addition to age and gender, there were several statistically significant associations (*p* = 0.029 to 0.048, η^2^ = 0.00 to 0.059) in a negative direction; decreasing scores on measures of g-factor and g-speed were associated with increasing RNFL thickness (*r = −*0.229 to −0.243, *p* < 0.05). No significant associations were found between RNFL thickness and g-memory ability. When we considered the number of years of education as a covariate, we found no significant associations between the RNFL thickness and cognitive scores.

**Conclusions:**

In a community dwelling cohort of healthy older people, increased RNFL thickness appeared to be associated with lower general processing speed and lower general cognitive ability when age 11 IQ scores were included as a covariate.

## Background

Cognitive ability refers to such things as the capacity of an individual to perform the higher mental processes such as reasoning, remembering, understanding, thinking quickly, and problem solving [1]. To work efficiently, complex cognitive functions require intact connections between distant brain regions that form neural networks [2,3]. Cognitive decline in the elderly population is one of the most feared aspects of growing old and can have significant impact on an individual’s quality of life as well as on the burden to society in caring for those affected. Cognitive decline occurs, on average, within some cognitive domains as people grow older from young adulthood, but the rate of decline varies between individuals [4]. The clinical determination of cognitive decline, especially in the milder spectrum is not easy although there has been some promising success with the use of biochemical and neuro-imaging markers as diagnostic aids [5]. Better diagnosis and discovering the determinants of individual differences in general and specific aspects of cognitive ageing would be important and logical initial steps in trying to improve the lives of older people. The eye and brain areas that are responsible for cognitive functioning share the same embryonic origins and it is possible that they both share common age-related features. Common pathogenic mechanisms such as inflammation and complement activation have been demonstrated by studies showing amyloid-beta deposition in retinal drusen in eyes with age-related macular degeneration and senile plaques in brains of the elderly or patients with Alzheimer’s disease (AD) [6,7]. AD represents an extreme spectrum of cognitive decline, but senile plaques occur widely in older adults who do not have dementia.

Two clinical studies have suggested an association between cognitive function and retinal ganglion cell loss as evidenced by a statistically significant decline in mean retinal nerve fiber layer (RNFL) thickness with mild cognitive impairment and AD compared to age-matched controls [8,9]. Moreover, AD has also been associated with retinal ganglion cell loss through histopathologic postmortem studies [8,10,11] and with a reduced RNFL thickness in two in-vivo studies [12,13]. The association of retinal morphological changes associated with retinal ganglion cell loss and cognitive function has also been supported by studies describing an increased prevalence of glaucoma in patients with AD [14,15]. Furthermore, in multiple sclerosis, the degree of RNFL atrophy has been shown to correlate significantly with cognitive disability [16].

These findings raise the possibility of RNFL thickness to be used as a biomarker of cognitive ageing. However, the association of RNFL and cognitive function in normal elderly population is not well established. Here we report the relationship between RNFL thickness and cognitive abilities in a narrow-age sample of 105 individuals, all born in Scotland in 1936, and about age 72 years when tested. RNFL thickness was measured using the Stratus Optical Coherence Tomography (OCT) (Carl Zeiss Meditec, UK). OCT has been used extensively in many previous studies to assess RNFL thickness in vivo in various ophthalmic and neurologic diseases [17-19], as well as neurodegenerative disorders with frequent cognitive changes like schizophrenia [20], obstructive sleep apnoea syndrome [21], or acute mountain sickness [22], and has been widely tested in the elderly. Thinning of the RNFL has been noted with ageing, as demonstrated not only by OCT in different populations [23-27], but also by red-free photography [28], scanning laser polarimetry [29] and nerve fibre counts of post mortem human tissue [30]. OCT uses interference patterns of backscattered near-infrared light and yields measurements (in micrometers) that are accurate to within 5 to 6 μm of histologic parameters [25,31], which could potentially assess the extent of neuronal death and axonal loss in RNFL [32]. We tested for the first time whether these easily-quantifiable properties of the retina could provide a biomarker of relative cognitive function status in older people without cognitive pathology.

## Methods

### Study population

The data were drawn from a sub-sample of the Lothian Birth Cohort (LBC) 1936 study whose recruitment and characteristics have been described in detail previously [33]. This consecutive series of unselected sub-sample (n = 105) underwent OCT testing performed by two ophthalmologists (AL and GL) at the Princess Alexandra Eye Pavilion, NHS Lothian, Scotland. Prior to the OCT measurement, the visual acuity was assessed, a dilated fundus examination performed and wide field colour fundus photographs were taken to exclude major eye diseases, such as retinal disorders or glaucoma, that could act as confounders. There were 61 males and 44 females in the sample who were screened for cognitive pathology using the Mini-Mental State Examination (MMSE) with scores above 24. They were all born in 1936 with a mean age of 73 (SD = 0.27) at the time of RNFL measurement. All the cognitive data were collected at a prior assessment (mean time elapsed between assessments = 381 days) at the Wellcome Trust Clinical Research Facility, Western General Hospital, NHS Lothian, Scotland. Of this group, 101 participants completed the Moray House Test (MHT)—an omnibus test of general intelligence with items predominantly based on verbal reasoning—at a mean age of 11 years as part of Scottish Mental Survey (SMS) of 1947. This test has a high concurrent validity with the Stanford-Binet test. The research adhered to the tenets of the Declaration of Helsinki and was approved by Lothian Research Ethics Committee. Written informed consent for participation in the study was obtained from all the participants.

### RNFL assessment

OCT assessment was done using the Fast RNFL scan protocol which acquired 256 measurements taken in a circle with a standardized diameter of 3.4 mm around the optic disc 3 times for a total of 768 data points per scan. The examiner centered the aiming circle around the optic disc each time and polarization was optimized to maximize the reflective signal. The acquired RNFL measurement was divided into four areas representing the superior, temporal, inferior and nasal quadrants. Each individual scan produced data for each quadrant and these were summed to produce an overall mean for each measurement.

### Cognitive function measures

At age ~72 years, participants completed several tests of cognitive ability; each test is described in detail in freely available resource [33]. A short description is given here. A number of the cognitive tasks were drawn from the Wechsler Adult Intelligence Scale-III^UK^ (WAIS-III, 1998): Matrix Reasoning (assesses nonverbal inductive reasoning), Block Design (assesses spatial and constructional abilities), Digit Symbol Coding and Symbol Search (which measure information processing speed) and Letter-number Sequencing and Backward Digit Span (which assess working memory). Three other measures of processing speed were also completed: simple mean reaction time (SRT), choice mean reaction time (CRT) and inspection time (IT). Reaction time parameters were measured using a custom-made standalone portable box. Inspection time is a computerized task used to assess elementary visual processing with no requirement for speeded reactions.

Several tests were also completed from the Wechsler Memory Scale- III^UK^ (WMS-III, 1998): Verbal Paired Associates (attempting to recall 8 phonologically and semantically unrelated word pairs) immediately and after ~30 minutes have elapsed from the immediate recall, Spatial Span (a nonverbal memory task) and Logical Memory (assesses verbal declarative memory). Letter-number Sequencing and Backward Digit Span appear in the WMS-II as well as the WAIS-III.

At age ~11 years participants completed the MHT, a paper and pencil general cognitive function test which must be completed within 45 minutes. There are 75 items on the test with a maximum score of 76. The raw scores on this test were corrected for ages in days of testing and then converted to an intelligence quotient (IQ) score scale (mean = 100, SD = 15).

### Statistical analyses

Using principal component analysis (PCA), a general cognitive ability component score (g-factor) was derived from six WAIS-III subtests (Letter-number sequencing, Matrix reasoning, Block design, Digit symbol, Digit Span backwards, Symbol search). A PCA of the set of speed measures (Symbol search, Digit symbol, Simple RT mean, Choice RT mean, Inspection time) yielded a general processing speed component score (g-speed). The extraction of these factors by PCA has been described elsewhere [34]. Using the same method, a general memory factor (g-memory) was extracted from the WMS-III subtests (Logical memory I immediate and II delayed recall, Spatial span forwards, Spatial span backwards, Verbal paired associates I immediate and II delayed recall) and two WAIS-III subtests (Letter-number sequencing, Digit Span backwards). The first un-rotated principal components for the g*-*factor, g-speed, and g-memory accounted for 51%, 53%, and 48% of the total relevant test variance, respectively.

## Results

Of the 105 subjects, we acquired 96 complete sets of RNFL measurements that were of sufficient quality to perform further analysis. The mean visual acuity (logMAR equivalent) was 0.11 (SD = 0.14; Range: 0.00 to 0.50). We included only participants who contributed complete cognitive data for the cognitive analyses; 3 participants were excluded from the RNFL-cognition analysis as they did not have age 11 MHT scores. Additionally, a selection of participants (n = 21) had the same eye measured twice to assess the repeatability of the retinal OCT measurements. Any points which fell more than 3 standard deviations above or below the mean were treated as outliers and excluded because extreme values can have a disproportionate influence on results. Six points in total were excluded across the superior, temporal and nasal areas.

### Inter-quadrant comparisons

The RNFL thickness in the four quadrants were similar to those reported in the literature, with the thicker superior quadrant (mean = 100.09 μm, SD = 16.09) and inferior quadrant (mean = 117.18 μm, SD = 20.04) and the thinner temporal quadrant (mean = 66.00 μm, SD = 14.27) and nasal quadrant (mean = 71.34 μm, SD = 15.39). We also found significant correlations between most of the four quadrants (Table 1). A PCA of these four areas yielded a factor which explained 54% of the variance. An equivalent left eye ‘global’ OCT parameter was calculated which accounted for 53% of the variance.

**Table 1:** Descriptive statistics and Pearson’s correlations between the four retinal quadrants

	**Mean (μm)**	**Standard deviation**	**Superior area**	**Temporal area**	**Inferior area**	**Nasal area**
Superior Area	100.09	16.09	1			
Temporal Area	66.0	14.27	.296 (.004)	1		
Inferior Area	117.18	20.04	.508 (.001)	.484 (.001)	1	
Nasal Area	71.34	15.39	.435 (.001)	-.005 (.960)	.460 (.001)	1

*Note.* N = 91-95.

All *p* values given in brackets.

### Left-right comparisons

We examined the relationship of these four quadrants and the global RNFL parameter, between both eyes to assess inter-ocular equivalency. Table 2 lists the intra-class correlation coefficients (ICC); all were significant at *p* < 0.05 level (ICCs = 0.38 - 0.74). We also used Bland Altman graphs [35] to examine the level of agreement between the RNFL measurements in the left and right eyes to determine whether there was any systematic measurement error. We found that the mean difference score between the RNFL of the 4 quadrants was close to zero and that most points fell on or around this line. There appeared to be no systematic measurement error and high levels of inter-ocular equivalency between the left and right eye measurements; therefore only right eye RNFL data was used in subsequent analyses.

**Table 2:** Left-Right descriptive statistics and intraclass correlation coefficients (ICC)

	**Right eye mean in μm (SD)**	**Left eye mean in μm (SD)**	**ICC single measures**	**ICC average measures**
Superior Area	100.09 **(16.09)**	99.88 **(15.73)**	.39 (.016)	.56 (.016)
Temporal Area	65.99 **(14.27)**	61.16 **(14.0)**	.60 (.001)	.74 (.001)
Inferior Area	117.18 **(20.04)**	114.63 **(21.40)**	.38 (.018)	.55 (.018)
Nasal Area	71.34 **(15.39)**	68.44 **(14.67)**	.47 (.005)	.64 (.005)
Global RNFL	0 **(1)**	0 **(1)**	.47 (.009)	.60 (.009)

Notes.

Exact *p* values given in brackets.

Standard deviations given in bracketed bold typeface.

N = 30

### Intra-rater and inter-rater reliability

We also analyzed a subset of the data which where RNFL thickness was measured twice. As the RNFL measurements were performed by two different examiners (AL and GL), we split the data to reflect the potential for this to be an additional confounding variable. ICC were also presented for the full data. A ‘global’ factor for this re-rated data was again computed using a PCA; the first unrotated factor accounted for 58% of the variance. The resultant analyses (Tables 3, 4) show high repeatability of the measure for the full (*p <*0.05, ICCs = 0.78 - 0.97) and split data (*p <*0.05, ICCs = 0.77 - 0.97). Bland Altman graphs were also used to assess agreement between separate ratings and we found high levels of agreement and no systematic measurement error between the 2 examiners.

**Table 3:** Descriptive statistics and intra-rater reliability for right eye measurements

	**Right eye mean in μm (SD)**	**Mean in μm (SD) rating 2**	**ICC single measures**	**ICC average measures**
Superior Area	100.09 **(16.09)**	100.82 **(22.45)**	.85 (.0001)	.92 (.001)
Temporal Area	65.99 **(14.27)**	64.82 **(17.09)**	.91 (.001)	.95 (.001)
Inferior Area	117.18 **(20.04)**	110.28 **(21.0)**	.94 (.001)	.97 (.001)
Nasal Area	71.34 **(15.39)**	69.27 **(15.44)**	.78 (.001)	.88 (.001)
Global RNFL	0 **(1)**	0 **(1)**	.93 (.001)	.97 (.001)

Exact *p* values given in brackets after ICCs.

Standard deviations given in bold typeface.

N = 21.

**Table 4:** Descriptive statistics and inter-rater reliability for right eye measurements

	**Rater A: right eye mean in μm (SD)**	**Rater A: mean in μm (SD) Rating 2**	**Rater A: ICC single measures**	**Rater A: ICC average measures**	**Rater B: right eye mean in μm (SD)**	**Rater B: mean in μm (SD) Rating 2**	**Rater B: ICC single measures**	**Rater B: ICC average measures**
Superior Area	101.68 **(17.80)**	101.42 **(23.79)**	0.92 (.001)	.96 (.001)	98.94 **(16.04)**	100.1 **(21.98)**	.78 (.002)	.88 (.002)
Temporal Area	64.48 **(14.69)**	59.56 **(13.65)**	.94 (.001)	.97 (.001)	67.14 **(13.97)**	71.13 **(19.30)**	.89 (.001)	.94 (.001)
Inferior Area	114.81 **(18.86)**	107.64 **(21.63)**	.95 (.001)	.98 (.001)	118.95 **(20.87)**	113.43 **(20.90)**	.92 (.001)	.96 (.001)
Nasal Area	71.08 **(14.4)**	67.83 **(14.53)**	.77 (.002)	.87 (.002)	71.55 **(16.25)**	71.0 **(17.09)**	.79 (.002)	.88 (.002)
Global RNFL	-.05 **(1.09)**	-.14 **(1.12)**	.95 (.001)	.97 (.001)	.04 **(.94)**	.17 **(.86)**	92 (.001)	.96 (.001)

exact *p* values given in brackets.

Standard deviations given in bold typeface.

n (rater A) = 11, n (rater B) = 10.

### RNFL and cognition models

We used general linear modeling to calculate the effect of RNFL thickness on the various general cognitive domains (g-factor, g-speed and g-memory) and IQ at age 11. Age in days at time of cognitive assessment and sex were used as co-variates in all analyses. We found only one statistically significant association, between the inferior area RNFL thickness and g-speed (*p* = .049, η^2^ = 0.45); all other associations between RNFL measurements and the cognitive measures were statistically non-significant (Table 5). In the next stage of analysis, the same general linear models were re-run including age 11 IQ as a covariate in addition to age and sex. In effect, this tested the association between RNFL thickness and cognitive change between 11 and 72, as opposed merely to cognitive status at age 72. We found several additional statistically significant associations (Table 5). To clarify the direction of effect, and to make effect sizes more accessible, we computed partial correlations for these associations and found that these were all in a negative direction; decreasing scores on measures of general cognitive ability and general processing speed were associated with increasing RNFL thickness (*r = −*0.229 to −0.243, *p* < 0.05) (data not shown).

**Table 5:** General Linear Models examining the relationship between RNFL thickness in each quadrant and cognitive outcomes at age ~72 (p values and partial eta squared values)

	**G-Factor**		**G-Speed**		**G-Memory**		**Age 11 IQ**	
	***P***	**η**^**2**^	***P***	**η**^**2**^	***P***	**η**^**2**^	***P***	**η**^**2**^
Superior Area	.089 **(.034)**	.006 **(.052)**	.141 (.093)	.026 (.034)	.321 (.294)	.012 (.013)	.924	.000
Temporal Area	.878 (.950)	.000 (.000)	.763 (.566)	.001 (.004)	.866 (.984)	.000 (.000)	.734	.001
Inferior Area	.063 **(.023)**	.040 **(.059)**	**.049 (.034)**	**.045 (.053)**	.660 (.646)	.002 (.002)	.905	.000
Nasal Area	.238 (.082)	.017 (.036)	.235 (.161)	.017 (.024)	.087 (.087)	.034 (.035)	.733	.001
Global RNFL	.126 **(.029)**	.030 **(.058)**	.103 **(.048)**	.034 **(.050)**	.225 (.270)	.018 (.015)	.757	.001

*Note.* All associations adjusted for age at time of cognitive assessment and sex.

^a^Associations given in brackets are controlled for age 11IQ (MHT scores at age 11 converted at an IQ type score) scores.

Statistically significant associations and their corresponding η^2^ are given in bold typeface.

N = 82–90.

## Discussion

We found that, in a community dwelling cohort of healthy older people, there were significant associations between RNFL thickness and certain types of cognitive function when age 11 IQ was included as a covariate. Increasing RNFL thickness was associated with lower general cognitive ability and general processing speed. There were no significant associations found with the general memory ability score. This finding differed from a recent population-based study by van Koolwijk et al. who found that, although there was an association between thicker RNFL and higher cognitive functioning in the younger population, this diminished to non-significance in those aged 40 and over [36]. They concluded that this lack of association in older individuals suggested that loss of neurons in the cerebrum and retina were not concomitant and might have different origins. In our study, RNFL thickness was also found to be a potential biomarker of relative cognitive status in the older age group, albeit in an unexpected direction, with a thicker RNFL being associated with a lower cognitive functioning. The reasons for this are not entirely clear, although different techniques were used to measure RNFL thickness in both studies (scanning laser polarimetry [36] as opposed to optical coherence tomography in our study). Moreover, in the study by van Koolwijk et al. the vast majority of participants (78%) had consanguineous parents and were derived from an isolated population, which may not necessarily represent the general population [36].

Also, although previous studies have shown a decreased peripapillary RNFL thickness in mild cognitive impairment and Alzheimer disease patients [9,13], these studies included only a small number of patients and no detailed cognitive function measures were employed [34], as in this study. Despite the proposed link between decreased RNFL thickness and cognitive impairment in a disease context, the Lothian Birth Cohort 1936 study suggests for the first time that this may not be the case in healthy individuals and that the link between parallel processes in RNFL and cognitive function may be more complex than previously thought, especially when considering not only cognitive function at the age of 72, but also lifetime cognitive change between 11 and 72. We can only speculate that changes in cognition in the ageing brain are a complicated phenomenon and one that may not be explained by the loss of neurons in the retina. The direction and magnitude of our findings would also need replicating in a different population.

A special strength of our study was the narrow chronological age range of the subjects. Most ageing biomarkers are strongly affected by chronological age. Therefore, it is helpful to omit that as far as possible when attempting to find the association between a putative biomarker and another variable, such as cognition. Otherwise, any biomarker-cognition association tends to reflect chronological age, something which is difficult to expunge, even when age is statistically adjusted. Another special strength of our study was the availability of the age 11 cognitive scores to test as a covariate which allowed us to examine the correlation of the RNFL to the phenotype of lifetime cognitive change. Another strength of our study was the careful determination of the reproducibility and repeatability of our RNFL measurements using the OCT, by using assessments of the ICC and levels of agreements between the left and right eyes and between the 2 testers. This ensured the exclusion of any systematic measurement errors.

The role of cognitive reserve in influencing the cognitive ability later in life was considered. The estimation of cognitive reserve is not very well established, with different proxies described in the literature, including variables descriptive of lifetime experiences (eg. educational attainment, income or social attainment) [37]. Premorbid intelligence (in our series, the age 11 IQ) is a powerful measure of cognitive reserve [38-41]. However, education and other life experiences may impart reserve over and above that obtained from childhood intelligence [42]. When we re-analysed our data to include the number of years of education as a variable, we found no significant associations between the RNFL thickness and cognitive scores.

Our study had some limitations. Firstly, we have not controlled for all potential confounders such as refractive error that might possibly affect the OCT measurements. Although it is known that myopia is associated with correlates of cognitive functioning such as intelligence and educational attainments, the prevalence of myopia is considered low in the elderly population [43]. The findings could also be due to type 1 error and further analysis on a larger sample size could help confirm the associations reported here between RNFL and cognitive function. Another limitation which might need further exploring relates to the possibility that cognitive ability might be influenced by cognitive reserve acquired for example by level and duration of education received which could in turn delay the onset of cognitive troubles while the brain lesions and biomarkers are progressing. In our study, we used the MMSE and relied on self-reporting by participants to exclude cognitive impairment and could have included those with mild cognitive impairment but with MMSE scores within the normal range. As shown by Paquet et al., subjects with mild cognitive impairment may have modified retinal thickness [9]. Another potential area for investigation would be to explore the relationship between the RNFL, cognitive function and other biomarkers including retinal vascular morphology features such as vessel caliber, tortuosity and branching geometry.

## Conclusions

In summary, we found that in a community dwelling cohort of healthy older people, increased RNFL thickness appeared to be associated with lower general processing speed and lower general cognitive ability when age 11 IQ scores were included as a covariate.

## Competing interests

The authors declare that they have no competing interests.

## Authors’ contributions

AL is responsible for overall patient recruitment and management of the project and drafted the manuscript. GL is responsible for patient recruitment and data collection. RDH, JMS, IJD and BD are responsible for the design of the study and the co-ordination of resources. All authors read, critically reviewed and approved the final manuscript.

## Pre-publication history

The pre-publication history for this paper can be accessed here:

http://www.biomedcentral.com/1471-2415/13/28/prepub
